# Zinc Oxide Nanoparticle Improves the Intestinal Function of Intrauterine Growth Retardation Finishing Pigs *via* Regulating Intestinal Morphology, Inflammation, Antioxidant Status and Autophagy

**DOI:** 10.3389/fvets.2022.884945

**Published:** 2022-06-06

**Authors:** Binbin Zhou, Jiaqi Zhang, Huijuan Liu, Shun Chen, Tian Wang, Chao Wang

**Affiliations:** College of Animal Science and Technology, Nanjing Agricultural University, Nanjing, China

**Keywords:** intrauterine growth retardation finishing pigs, zinc oxide nanoparticle, immunity, inflammation, antioxidant capacity, autophagy

## Abstract

This study was to investigate effects of zinc oxide nanoparticle (Nano-ZnO) on growth, immunity, intestinal morphology and function of intrauterine growth retardation (IUGR) finishing pigs. Six normal birth weight (NBW) and 12 IUGR male piglets were obtained and weaned at 21 d. NBW-weaned piglets fed basal diets (NBW group), IUGR-weaned piglets allocated to two groups fed basal diets (IUGR group) and basal diets further supplemented 600 mg Zn/kg from Nano-ZnO (IUGR+Zn group), respectively. All pigs were slaughtered at 163 d. Results showed: (1) IUGR pigs showed no difference in body weight at 77d and 163d (*P* > 0.05), while had increased villus height (VH) and villus surface area in jejunum (*P* < 0.05) and enhanced interleukin-6, TNF-α and NF-κB mRNA expression (*P* < 0.05) as compared to NBW group; Compared with IUGR group, dietary Nano-ZnO did not affect the body weight (*P* > 0.05), but increased VH to crypt depth ratio and IgA concentration (*P* < 0.05) and decreased TNF-α and NF-κB mRNA expression in jejunum (*P* < 0.05). (2) IUGR increased the number of swollen mitochondria and autolysosomes, and protein expressions of sequestosome-1 (P62) and microtubule-associated protein light chain 3 B/A (LC3B/A) in jejunum as compared to NBW group (*P* < 0.05); Compared with IUGR group, Nano-ZnO decreased the number of swollen mitochondria and autolysosomes, and P62 and LC3B/A protein expression (*P* < 0.05). (3) IUGR increased mucosal contents of malondialdehyde and protein carbonyl (PC) and Keap1 protein expression (*P* < 0.05) as compared to NBW group; Compared with IUGR group, dietary Nano-ZnO increased activities of total antioxidant capacity, catalase, glutathione peroxidase, and glutathione content (*P* < 0.05), and enhanced nuclear respiratory factor 2 (Nrf2), glutamate-cysteine ligase modifier subunit and glutathione peroxidase 1 mRNA expression, and increased total and nuclear Nrf2 protein expression (*P* < 0.05), and decreased malondialdehyde and PC content, and Keap1 protein expression (*P* < 0.05) in jejunum. Results suggested that IUGR pigs showed postnatal catch-up growth and improved intestinal morphology, and dietary Nano-ZnO may further improve intestinal morphology, reduce inflammation, decrease autophagy and alleviate oxidative stress via Nrf2/Keap1 pathway in jejunum of IUGR pigs.

## Introduction

Intrauterine growth retardation (IUGR) is a severe obstetric syndrome mainly caused by a fetal lack of nutrition or oxygen supply in the womb during pregnancy resulting in the hindrance of fetal growth and development *in utero* (1, 2). The incidence of IUGR in human fetuses is about 5%, and patients with IUGR usually have insulin resistance in the clinic, and may subsequently develop metabolic syndrome, such as type 2 diabetes mellitus and obesity, in adulthood (3–5). The natural incidence of IUGR in pigs can be as high as 15–20% in modern highly intensive farming patterns (6), and IUGR results in a low survival rate and has a significant negative impact on the growth performance of the piglets. However, IUGR pigs may show postnatal catch-up growth in body weight, but with oxidative stress, inflammation, obesity, and a higher risk of cardiovascular disease (7–9).

The jejunum is the principal place for nutrient digestion and absorption, and it is also one of the main organs of the body to participate in immune and inflammatory responses. Many studies have reported that IUGR can damage the intestinal morphology, reduce the immune ability and cause inflammation, and decrease in intestinal weight, villus height (VH), and the villus height to crypt depth ratio (VH/CD) in piglets, as well as increase crypt depth (CD) (10, 11). IUGR can also cause intestinal inflammation and decrease immunity, and induce oxidative stress in growing pigs (12). However, few studies have examined the effects of IUGR on the immune status and intestinal function of finishing pigs, despite the urgency of finding effective nutritional guidelines for IUGR pigs.

One nutrient that can maintain normal life activities of animals and is indispensable for all living organisms is zinc, a mineral element that needs to be consumed regularly to keep the animal healthy (13). The absorption rate of ordinary zinc oxide added to pig feed is very low, and most of the dietary zinc oxide will be excreted in the feces, with potential environmental pollution issues and resource waste (14). The rise of nanotechnology has led to the emergence of a variety of nanomaterials, including those containing zinc, in a historic moment. Zinc oxide nanoparticles (Nano-ZnOs) have high biological activity, antibacterial action and antioxidant properties and may be used as a source of zinc in feed. As the name implies, the size of Nano-ZnO is in the range of 1–100nm, and this size affects the physical and chemical properties through surface effects, quantum size effects, volume effects and macroscopic tunneling effects (15). Dietary supplementation of 400–800 mg/kg Nano-ZnO can improve growth performance, reduce diarrhea and regulate the intestinal microbiota of weaned piglets, and show similar positive effects to 3,000 mg/kg traditional zinc oxide (16, 17). The beneficial effects of dietary 1,200 mg/kg Nano-ZnO on weaned piglets are similar to those of high dose of traditional zinc oxide plus antibiotics (3000 mg/kg ZnO + 20 mg/kg colistin sulfate) (18). Therefore, Nano-ZnO shows substantial promise for applications in the animal husbandry industry.

Given these anticipated potential positive effects of Nano-ZnO, we speculated that Nano-ZnO might show beneficial effects in IUGR pigs. Therefore, this study was conducted to investigate the effects of Nano-ZnO on the growth, immune status, jejunal morphology, antioxidant status, inflammation-related mRNA expression, mitochondrial ultrastructure, and autophagy in IUGR pigs.

## Materials and Methods

This study was approved and conducted under the supervision of the Institutional Animal Care and Use Committee of Nanjing Agricultural University (Jiangsu, China. Permit number SYXK-2019-00142).

### Experimental Animals, Diets and Design

The NBW and IUGR piglets were selected according to previous studies (19, 20). A total of 18 male piglets [Duroc × (Landrace × Yorkshire)] were selected from 6 sows with similar parity and litter size. The 6 NBW piglets (1.52 ± 0.01 kg) were separated into an NBW group and the 12 IUGR piglets (0.96 ± 0.02 kg) were randomly assigned to an IUGR group and an IUGR+Zn group. All piglets were weaned at 21 days of age and piglets in the NBW group and the IUGR group were fed a basal diet, whereas piglets in the IUGR+Zn group was fed the same basal diet supplemented with 600 mg Zn/kg from Nano-ZnO. Each treatment had six replicates (pens), and each replicate had 1 piglet. The effective supplemental level of Nano-ZnO was selected according to our previous study (16). The Nano-ZnO (95% purity) used in present study was the same as that used in our previous study (16) and was provided by Zhangjiagang Bonded Area Hualu Nanometer Material Co., Ltd. (Jiangsu, China).

After weaning, all piglets were fed in single slatted-floor cages at the same temperature (nursery period temperature was 26–30°C, fattening period was 20–24°C) and humidity (50–60%). Pigs were provided *ad libitum* access to feed and water throughout the trial period. Water supply, cage temperature and humidity, and the pig's physiology were checked twice a day. The piggery was cleaned and disinfected regularly, and the usual immunization program was maintained. The weight of each pig at days 1, 21, 77, and 163 was recorded, and the feed intake of each pig was accurately recorded to calculate the average daily gain (ADG), average daily feed intake (ADFI) and the gain-to-feed ratio (G:F) during 21 to 163 d.

### Sample Collection

After fasting for 12 h at the end of the feeding period (at 163 days of age), blood samples were obtained from each pig's precaval vein and centrifuged at 3,000 g for 10 min at 4°C to collect serum, which was then stored at −80°C for analysis. Subsequently, the pigs were euthanized by electrical stunning and exsanguinated. The jejunum samples (near the midpoint of jejunum) were collected from each pig for intestinal morphology analysis (fixed in 4% paraformaldehyde) and transmission electron microscope index (fixed in 2.5% glutaraldehyde solution), respectively. The jejunal mucosa was scraped, quickly frozen in liquid nitrogen and then stored at −80°C for further analysis.

### Serum Parameters of Hormones and Immunoglobins

The content of serum growth hormone (GH, MM-039701), insulin like growth factor 1 (IGF-1, MM-259701), immunoglobulin A (IgA, MM-090501), immunoglobulin G (IgG, MM-040301), immunoglobulin M (IgM, MM-040201) were determined via the enzyme-linked immunosorbent assay (ELISA) kits purchased from Jiangsu MeiMian Industrial Co., LTD (Jiangsu, China).

### Histological Morphology and Transmission Electron Microscopy of the Jejunum

The jejunal morphology was analyzed according a previous method reported by Dong et al. (21). Briefly, the fixed jejunum in 4% paraformaldehyde was dehydrated and impregnated with wax to prepare the section in thickness of 5 μm, and stained with hematoxylin and eosin (HE). All sections were captured by optical binocular microscope (Olympus BX5, Olympus Optical Co. Ltd, Japan) and digital camera (Nikon H550L). The VH, CD and villous width (VW) were measured with Image-Pro Plus soft-ware. The VH/CD and villous surface area (VSA) were calculated.

The fixed jejunum sample in 2.5% glutaraldehyde solution (24 h, 4°C) was further fixed in 1% osmium for 1 h, dehydrated with a series of hierarchical ethanol solutions and then fixed in epoxy resin. The sections were cut with ultrafine slicing machine (Boeckeler, Tucson, AZ, USA), photographed using a transmission electron microscope (Hitachi H-7650, Tokyo, Japan). The swollen mitochondria and autolysosome number was analyzed according to the methods in previous studies (22–24).

### Antioxidant and Immune Status of Jejunal Mucosa

With corresponding commercial kits purchased from Nanjing JianCheng Bioengineering Institute (Jiangsu, China), the concentrations of malondialdehyde (MDA, A003-1-2), protein carbonyl (PC, A087-1), total protein (A045-3-2), and glutathione (GSH, A005-1-2), as well as the activities of total antioxidant capacity (T-AOC, A015-2-1), catalase (CAT, A007-1-1), total superoxide dismutase (T-SOD, A001-1-1) and glutathione peroxidase (GSH-Px, A005-1-2) were determined. T-AOC, SOD, CAT, and GSH-Px activities were expressed in units (U) per milligram protein. MDA and PC concentrations were expressed in nanomole per milligram of protein. The GSH concentration was expressed in milligrams per gram of protein. The contents of IgG and IgA in intestinal mucosa were determined with corresponding commercial kits (Jiangsu MeiMian Industrial Co., Ltd, Jiangsu, China).

### RNA Extraction and Quantitative Real-Time Polymerase Chain Reaction

With the previous methods (16), we extracted the total RNA from jejunal mucosa, obtained the cDNA samples, and then reverse transcription polymerase chain reaction (RT-PCR) and the 2^−ΔΔ*Ct*^ method (25) were used to determine the relative mRNA expression. The specific primers of selected genes used in present study have been listed in Table 1, which are related to the inflammation [interleukin-1β (IL-1β), interleukin-4 (IL-4), interleukin-6 (IL-6), interleukin-10 (IL-10), tumor necrosis factor-α (TNF-α), interferon-γ (IFN-γ), nuclear factor kappa-B (NF-κB)], and antioxidant capacity [nuclear respiratory factor 2 (Nrf2), kelch like ECH associated protein 1 (Keap1), glutamate-cysteine ligase catalytic subunit (GCLC), glutamate-cysteine ligase modifier subunit (GCLM), superoxide dismutase 1 (SOD1), glutathione peroxidase 1 (GPx1)]. These primers were used in our previous studies (9, 12, 16), or designed and synthesized in a commercial company (Beijing Qingke Biological Technology Co., Ltd., Beijing, China). The β-actin was used as an internal reference gene (12, 20).

**Table 1:** Primer sequences used in quantitative real-time PCR assays.

**Name**	**Accession No**.	**Sequence (5' to 3')**	**Size (bp)**
Nrf2	NM_001114671.1	F: GACAAACCGCCTCAACTCAG	183
		R: GTCTCCACGTCGTAGCGTTC	
Keap1	XM_021076667.1	F: CGTGGAGACAGAAACGTGGA	239
		R: CAATCTGCTTCCGACAGGGT	
GCLC	XM_003482164.4	F: GGCGACGAGGTGGAATACAT	123
		R: GTTTGGGTTTGTCCTTTCCCC	
GCLM	XM_001926378.4	F: GCATCTACAGCCTTACTGGGA	180
		R: GTTAAATCGGGCGGCATCAC	
HO-1	NM_001004027.1	F: CAAGCAGAAAATCCTCGAAG	241
		R: GCTGAGTGTCAGGACCCATC	
SOD1	NM_001190422.1	F: CATTCCATCATTGGCCGCAC	118
		R: TTACACCACAGGCCAAACGA	
GPx1	NM_214201.1	F: CCTCAAGTACGTCCGACCAG	85
		R: TGAGCATTTGCGCCATTCA	
IL-4	NM_214123.1	F: ACACGACGGAGAAGGAAACC	189
		R: GTTCCTGTCAAGTCCGCTCA	
TNF-α	NM_214022.1	F: ATCGGCCCCCAGAAGGAAGAG	351
		R: GATGGCAGAGAGGAGGTTGAC	
IL-6	NM_214399.1	F: AAATGTCGAGGCTGTGCAGA	207
		R: CTCAGGCTGAACTGCAGGAA	
IL-1β	NM_214029.1	F: TGCCAGCTATGAGCCACTTCC	337
		R: TGACGGGTCTCGAATGATGCT	
IL-10	NM_214041.1	F: GCATCCACTTCCCAACCA	446
		R: CTTCCTCATCTTCATCGTCAT	
IFN-γ	AY188090.1	F: TCAGCTTTGCGTGACTTTGTG	251
		R: GCTCTCTGGCCTTGGAACAT	
NF-κB	NM_001048232.1	F: GGCTACCCTGGCACAGAAAT	231
		R: GCCTGAGAGGTGGTCTTCAC	
β-actin	XM_003124280.4	F: CACGCCATCCTGCGTCTGGA	380
		R: AGCACCGTGTTGGCGTAGAG	

*Nrf2, nuclear respiratory factor 2; GCLC, glutamate-cysteine ligase catalytic subunit; GCLM, glutamate-cysteine ligase modifier subunit; HO-1, heme oxygennase-1; SOD1, superoxide dismutase 1; GPx1, glutathione peroxidase 1; IL-4, interleukin-4; TNF-α, tumor necrosis factor-α; IL-6, interleukin-6; IL-1β, interleukin-1β; IL-10, interleukin-10; IFN-γ, interferon-γ; NF-κB, nuclear factor kappa-B*.

### Western Blotting

The western blot analysis in jejunal mucosa was conducted according to previous studies (19, 26). The primary antibodies including Nrf2 (1:1,500), Keap1 (1:6,000), and P62 (1:4,000) were provided by Proteintech Group, Inc., (Rosemont, USA) and antibodies including microtubule-associated protein light chain 3 (LC3, 1:1,000) and β-actin (1:1,000) were provided by Cell Signaling (Danvers, Massachusetts, USA). The secondary antibody (1:5,000; Proteintech Group, Inc., Rosemount, IL, USA) was horseradish peroxidase labeled goat anti-rabbit immunoglobulin G in present study. The β-actin was used as the internal reference protein. For the protein expression of nuclear Nrf2, the protein used for western blot analysis was first extracted from the nuclei of jejunal mucosa with the nuclear extraction kit (SN0020, Beijing Solarbio Science&Technology Co., Ltd, China).

### Statistical Analysis

The data were processed with SPSS software (SPSS, ver. 22.0 for Windows, SPSS Inc., Chicago, USA), and statistical differences between groups were assessed using one-way analysis of variance and Tukey's *post-hoc* test for pairwise comparison. The data is presented as means and standard error of the mean (SEM), with a *P*-value of <0.05 indicating a statistically significant difference.

## Results

### Growth Performance

As shown in Table 2, body weights of pigs in the IUGR group were significantly lower at 1 d and 21 d (*P* < 0.05), but showed no significant difference at 77d and 163 d as compared with the NBW group (*P* > 0.05); body weights of pigs in the IUGR+Zn group were not significant different from that in the IUGR group (*P* > 0.05), but were significantly lower than that in the NBW group during this whole feeding experiment (*P* < 0.05). The ADG of pigs in the IUGR and IUGR+Zn group was lower than that of the NBW group during 1 to 21d (*P* < 0.05). During 21 to 77 d and 77 to 163 d, no significant differences in ADG, ADFI and G:F were observed between the IUGR and NBW groups (*P* > 0.05). Compared to the IUGR group, dietary 600 mg from Zn/kg Nano-ZnO did not affect the ADG, and G:F (*P* > 0.05), but significantly decreased ADFI of IUGR pigs during 77 to 163 d (*P* < 0.05). Moreover, pigs in the IUGR+Zn group had significantly lower ADG and ADFI during 77 to 163 d than that of the NBW group (*P* < 0.05).

**Table 2:** Effects of dietary Nano-ZnO supplementation on growth performance of IUGR finishing pigs.

**Items** ^**1**^	**Group** ^2^			**SEM**	***P*** **-value**
	**NBW**	**IUGR**	**IUGR+Zn**		
**BW (Kg)**					
1 d	1.52^a^	0.96^b^	0.96^b^	0.06	<0.001
21 d	6.06^a^	4.88^b^	4.86^b^	0.16	<0.001
77 d	30.93^a^	27.53^ab^	26.16^b^	0.83	0.042
163 d	121.83^a^	113.63^ab^	108.54^b^	1.82	0.003
**ADG (Kg / d)**					
1–21 d	0.22^a^	0.19^b^	0.19^b^	0.01	0.018
21–77 d	0.44	0.40	0.38	0.01	0.130
77–163 d	1.06^a^	1.00^ab^	0.96^b^	0.02	0.028
**ADFI (Kg / d)**					
21–77 d	0.87	0.78	0.72	0.03	0.114
77–163 d	2.53^a^	2.44^a^	2.16^b^	0.05	0.002
**G: F**					
21–77 d	0.52	0.52	0.55	0.03	0.746
77–163 d	0.42	0.41	0.45	0.01	0.220

1 *BW, body weight; ADG, average daily gain; ADFI, average daily feed intake; G: F, gain-to-feed ratio. Data were expressed as mean and SEM, n = 6;*

a, b *Means that values within a row with different superscript letters were significantly different (P < 0.05).*

2 *NBW, normal birth weight pigs; IUGR, intrauterine growth restriction pigs; IUGR+Zn, IUGR pigs fed with diets supplemented with 600 mg Zn/kg from Nano-ZnO*.

### Jejunal Morphology and Mitochondrial Ultrastructure

**Figure 1:**
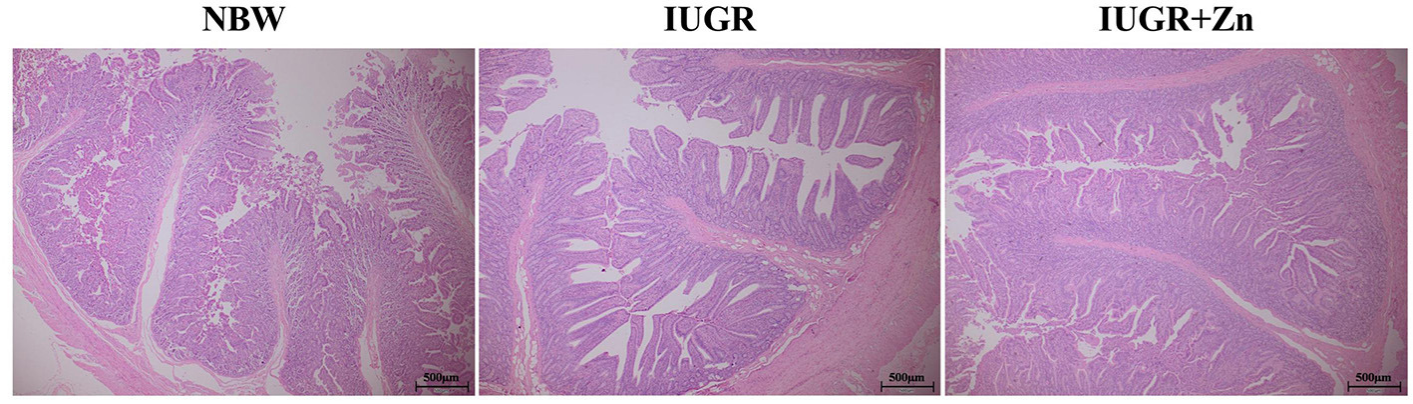
Effects of dietary Nano-ZnO supplementation on the jejunal histomorphology in IUGR finishing pigs. All samples were stained with hematoxylin and eosin (H&E). Scale bar represents 500 μm. NBW, images from normal birth weight pigs; IUGR, images from intrauterine growth restriction pigs; IUGR+Zn, images from IUGR pigs fed with diets supplemented with 600 mg Zn/kg from Nano-ZnO.

As shown in Table 3 and Figure 1, pigs in the IUGR group showed significantly higher VH, CD and VSA (*P* < 0.05), but had no significant difference in VW and VH/CD as compared to the NBW group (*P* > 0.05). Compared with the IUGR group, dietary added 600 mg Zn/kg from Nano-ZnO significantly lowered CD and increased VH/CD (*P* < 0.05), but did not affect the VH and VW of jejunum (*P* > 0.05). Compared to the NBW group, pigs in IUGR+Zn group had significantly higher VH, VH/CD and VSA (*P* < 0.05), and showed no significant difference in VW and CD (*P* > 0.05).

**Table 3:** Effects of dietary Nano-ZnO supplementation on jejunal morphology of IUGR finishing pigs.

**Items** ^**1**^	**Group** ^**2**^			**SEM**	***P*** **-value**
	**NBW**	**IUGR**	**IUGR+Zn**		
VH (μm)	416.14^b^	521.52^a^	582.81^a^	20.42	<0.001
VW (μm)	153.67	150.25	144.20	1.93	0.126
CD (μm)	223.46^b^	271.33^a^	233.86^b^	7.30	0.009
VH/CD	1.86^b^	1.94^b^	2.51^a^	0.09	0.001
VSA(mm^2^)	0.103^b^	0.124^a^	0.133^a^	0.004	0.004

1 *VH, Villus height; VW, Villus width; CD, Crypt depth; VH / CD, villus height to crypt depth ratio; VSA, Villus surface area. Results are expressed as mean and SEM, n = 6.*

a, b *Means that values within a row with different superscript letters were significantly different (P <0.05)*.

2 *NBW, normal birth weight pigs; IUGR, intrauterine growth restriction pigs; IUGR+Zn, IUGR pigs fed with diets supplemented with 600 mg Zn/kg from Nano-ZnO*.

**Figure 2:**
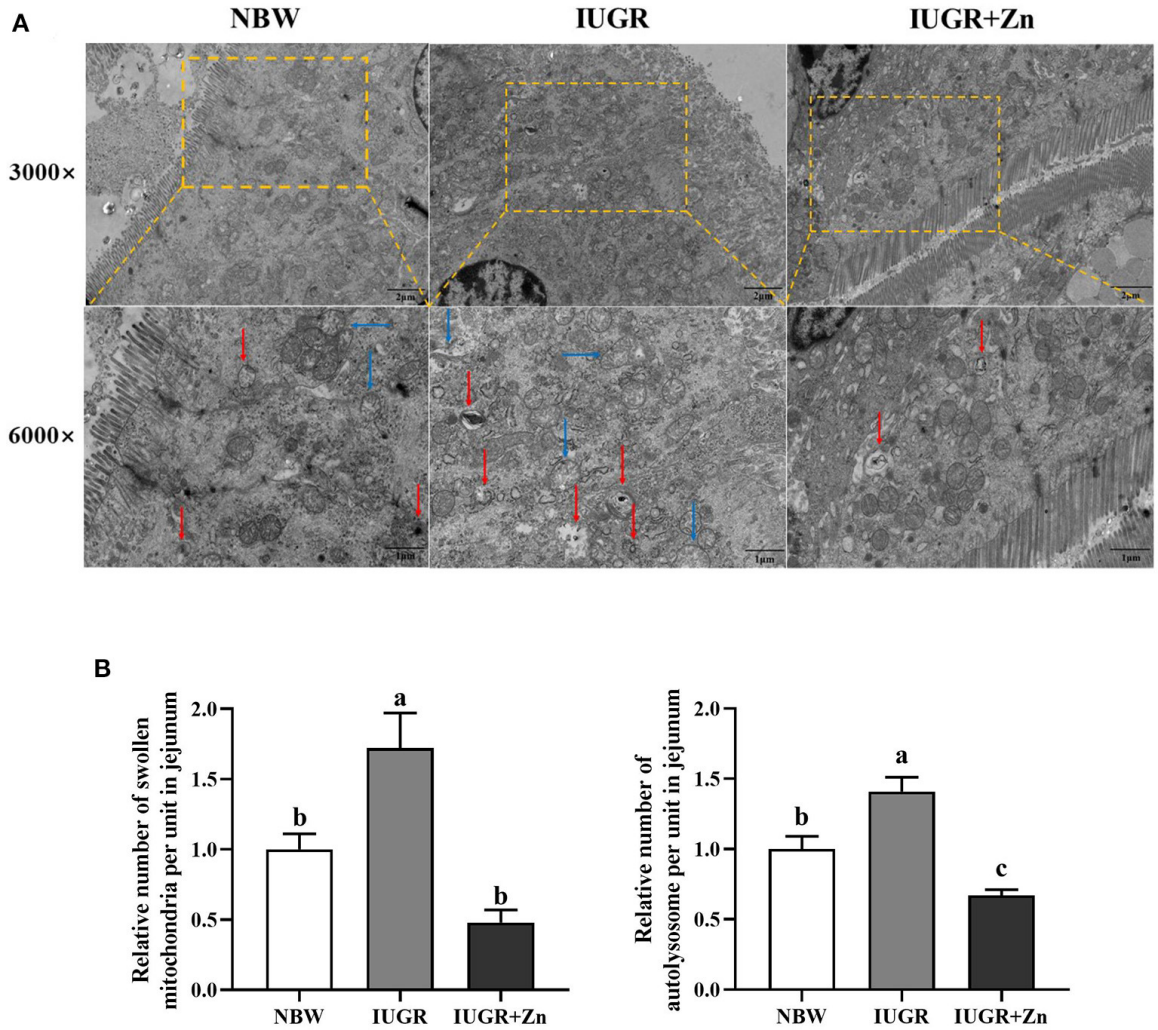
Effects of dietary Nano-ZnO supplementation on the jejunal mitochondrial ultrastructure of IUGR finishing pigs. **(A)** Representative image of jejunum using transmission electron microscopy, red arrows indicated autolysosome, blue arrows indicated the swollen mitochondria. **(B)** Quantitative analysis of swollen mitochondria and autolysosome of pigs' jejunal mucosa under transmission electron microscopy images (based on 3,000 times magnification, analysis of 4 images in each group, *n* = 4). Results were normalized by the mean value for the NBW group set to 1 unit. ^a, b^Means for the same parameter with different superscripts are significantly different (*P* < 0.05). NBW, normal birth weight pigs; IUGR, intrauterine growth restriction pigs; IUGR+Zn, IUGR pigs fed with diets supplemented with 600 mg Zn/kg from Nano-ZnO.

As shown in Figure 2, the mitochondria of jejunal enterocytes in the NBW group were clear, intact, and regular cristae. By contrast, the IUGR pigs had quite a few abnormal mitochondria in morphology, with poor integrity, and significantly increased the number of swollen mitochondria and autolysosomes (*P* < 0.05). However, compared with the IUGR group, dietary added 600 mg Zn/kg from Nano-ZnO effectively improved mitochondrial abnormalities, decreased the number of swollen mitochondria and autolysosomes in jejunal enterocytes of IUGR pig (*P* < 0.05). Moreover, pigs in the IUGR+Zn group showed significantly reduced number of autolysosomes (*P* < 0.05), but no significant differences in number of swollen mitochondria (*P* > 0.05) as compared to pigs in the NBW group.

### Hormones Parameters and Immune Status of Serum

No significant changes were obtained in concentrations of IGF-1, GH, IgA, IgG and IgM among the NBW, IUGR and IUGR+Zn groups (*P* > 0.05, Table 4).

**Table 4:** Effects of dietary Nano-ZnO supplementation on serum hormones parameters and immune status of the IUGR finishing pigs.

**Items** ^**1**^	**Group** ^**2**^			**SEM**	***P-*** **value**
	**NBW**	**IUGR**	**IUGR+Zn**		
IGF-1 (ng/mL)	753.48	740.35	753.56	6.26	0.639
GH (ng/mL)	22.57	22.79	22.76	0.42	0.976
IgG (mg/mL)	19.35	20.10	19.56	0.25	0.461
IgM (mg/mL)	15.46	15.93	15.47	0.23	0.677
IgA (μg/mL)	606.38	624.19	598.54	14.36	0.778

1 *IGF-1, insulin like growth factor1; GH, growth hormone; IgG, immunoglobulin G; IgM, immunoglobulin M; IgA, immunoglobulin A. Results are expressed as mean and SEM, n = 6.*

2 *NBW, normal birth weight pigs; IUGR, intrauterine growth restriction pigs; IUGR+Zn, IUGR pigs fed with diets supplemented with 600 mg Zn/kg from Nano-ZnO*.

### Antioxidant and Immune Status of Jejunal Mucosa

As shown in Table 5, IUGR finishing pigs exhibited significantly increased MDA and PC content (*P* < 0.05), but no significant difference in CAT, T-AOC, T-SOD, GSH-Px activity and GSH level in the jejunal mucosa compared with pigs in the NBW group (*P* > 0.05). Compared to the IUGR group, dietary added 600 mg Zn/kg from Nano-ZnO significantly lowered the MDA and PC content (*P* < 0.05) and enhanced the CAT, T-AOC, GSH-Px activity and GSH content (*P* < 0.05) in the jejunal mucosa of IUGR pigs, but did not significantly alter the T-SOD activity (*P* > 0.05).

**Table 5:** Effects of dietary Nano-ZnO supplementation on the antioxidant status of jejunal mucosa of the IUGR finishing pigs.

**Item** ^**1**^	**Group** ^**2**^			**SEM**	***P*** **-value**
	**NBW**	**IUGR**	**IUGR+Zn**		
MDA (nmol/mgprot)	0.46^b^	0.75^a^	0.48^b^	0.04	0.001
PC (nmol/mgprot)	13.52^b^	18.66^a^	12.41^b^	0.82	<0.001
CAT (U/mgprot)	29.81^ab^	23.44^b^	34.32^a^	1.76	0.028
T-AOC (U/mgprot)	0.26^b^	0.24^b^	0.49^a^	0.03	<0.001
GSH (mg/gprot)	5.45^b^	3.91^b^	9.63^a^	0.70	<0.001
T-SOD (U/mgprot)	262.87	251.84	263.20	12.70	0.926
GSH-Px (U/mgprot)	91.96^b^	90.21^b^	102.69^a^	1.90	0.006

1 *MDA, malondialdehyde; PC: Protein carbonyl. CAT, catalase; T-AOC, total antioxidant capacity; GSH, glutathione, T-SOD, total superoxide dismutase; GSH-Px, glutathione peroxidase. Data are expressed as mean and SEM, n = 6*.

a, b *Means that values within a row with different superscript letters were significantly different (P <0.05)*.

2 *NBW, normal birth weight pigs; IUGR, intrauterine growth restriction pigs; IUGR+Zn, IUGR pigs fed with diets supplemented with 600 mg Zn/kg from Nano-ZnO*.

Results of Table 6, showed that no significant changes in mucosal concentration of IgG among the NBW, IUGR, and IUGR+Zn groups (*P* > 0.05), while dietary added 600 mg Zn/kg from Nano-ZnO significantly decreased the IgA content in jejunal mucosa of IUGR pigs (*P* < 0.05). No significant change in IgA in jejunal mucosa was obtained between the NBW and IUGR+Zn group (*P* > 0.05).

**Table 6:** Effects of dietary Nano-ZnO supplementation on immune status of jejunal mucosa in IUGR finishing pigs.

**Items** ^**1**^	**Group** ^**2**^			**SEM**	***P*** **-value**
	**NBW**	**IUGR**	**IUGR+Zn**		
IgG (μg/mgprot)	130.33	133.36	119.30	2.67	0.069
IgA (μg/mgprot)	13.75^ab^	15.14^a^	13.01^b^	0.34	0.024

1 *IgG, immunoglobulin G; IgM, immunoglobulin M; IgA, immunoglobulin A. Results are expressed as mean and SEM, n = 6*.

a, b *Means that values within a row with different superscript letters were significantly different (P <0.05)*.

2 *NBW, normal birth weight pigs; IUGR, intrauterine growth restriction pigs; IUGR+Zn, IUGR pigs fed with diets supplemented with 600 mg Zn/kg from Nano-ZnO*.

### MRNA Expression

**Figure 3:**
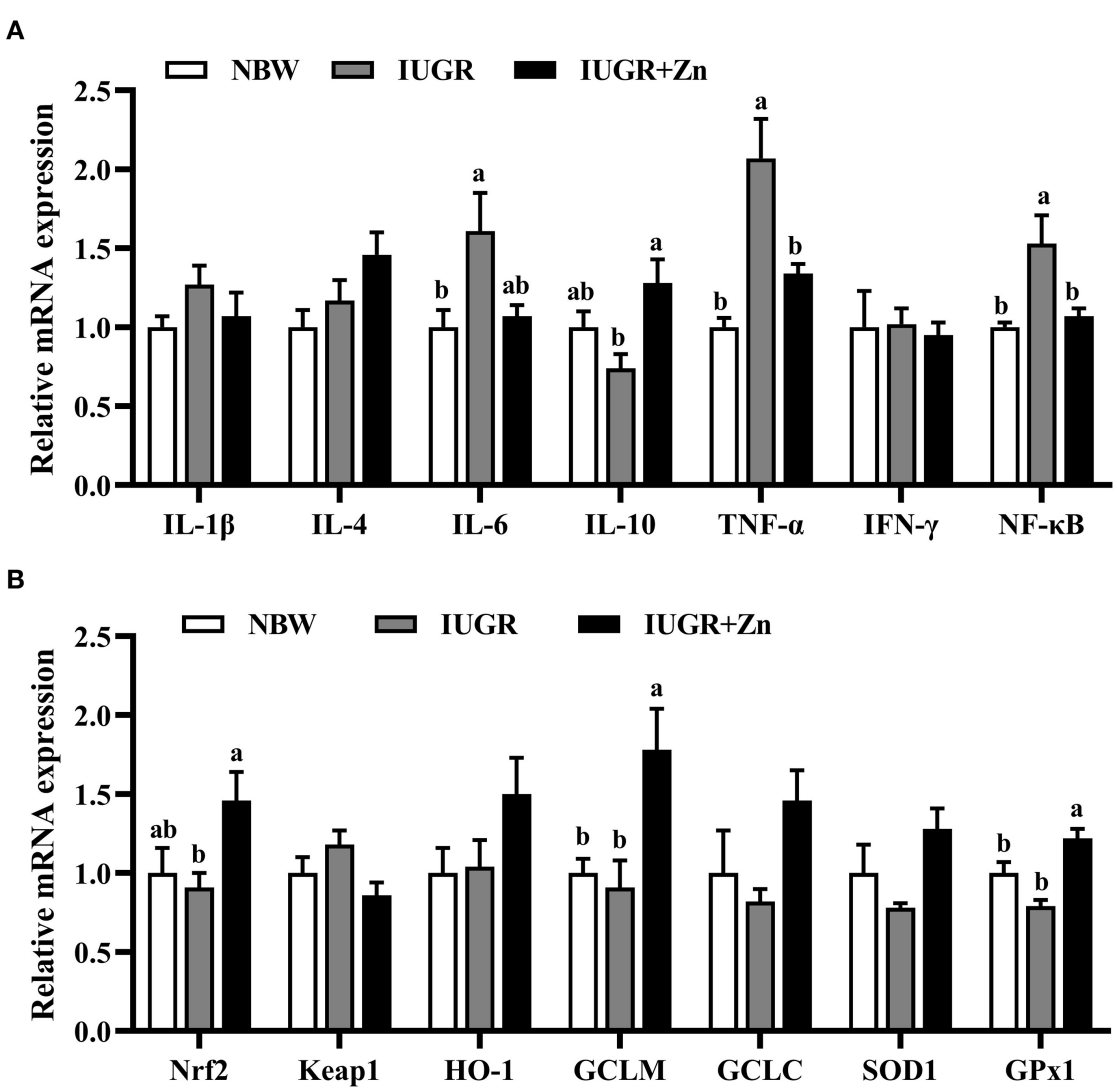
Effects of dietary Nano-ZnO supplementation on the mRNA expressions in jejunal mucosa of finishing pigs. **(A)** Inflammation related mRNA expression. **(B)** Antioxidant related mRNA expression. Data are normalized to the NBW group and expressed as means with their SEM (*n* = 6). ^a, b^Means for the same parameter with different superscripts are significantly different (*P* < 0.05). IL-1β, interleukin-1β; IL-4, interleukin-4; IL-6, interleukin-6; IL-10, interleukin-10; TNF-α, tumor necrosis factor-α; IFN-γ, interferon-γ; NF-κB, nuclear factor kappa-B; Nrf2, nuclear respiratory factor 2; GCLC, glutamate-cysteine ligase catalytic subunit; GCLM, glutamate-cysteine ligase modifier subunit; SOD1, superoxide dismutase 1; GPx1, glutathione peroxidase 1. NBW, normal birth weight pigs; IUGR, intrauterine growth restriction pigs; IUGR+Zn, IUGR pigs fed with diets supplemented with 600 mg Zn/kg from Nano-ZnO.

As showed in Figure 3, compared with the NBW group, IUGR finishing pigs had significantly enhanced mRNA expression of IL-6, TNF-α and NF-κB (*P* < 0.05), and no significant differences in the mRNA expression of Nrf2, Keap1, HO-1, GCLC, GCLM, SOD1, GPx1, IL-1β, IL-4, IL-10 and IFN-γ (*P* > 0.05). Compared with the IUGR group, dietary added 600 mg Zn/kg from Nano-ZnO significantly enhanced the mRNA expression of Nrf2, GCLM, GPx1 and IL-10 (*P* < 0.05), and reduce the mRNA expression of TNF-α and NF-κB in jejunal mucosa of IUGR pigs (*P* < 0.05), while did not significantly alter the mRNA expression of Keap1, HO-1, GCLC, SOD1, IL-1β, IL-4, IL-6 and IFN-γ (*P* > 0.05). The mRNA expression of GCLM and GPx1 significantly enhanced in the IUGR+Zn group (*P* < 0.05) as compared with the NBW group, while no significant difference in the other selected antioxidant and inflammation related mRNA expression was observed (*P* > 0.05).

### Proteins Expression

**Figure 4:**
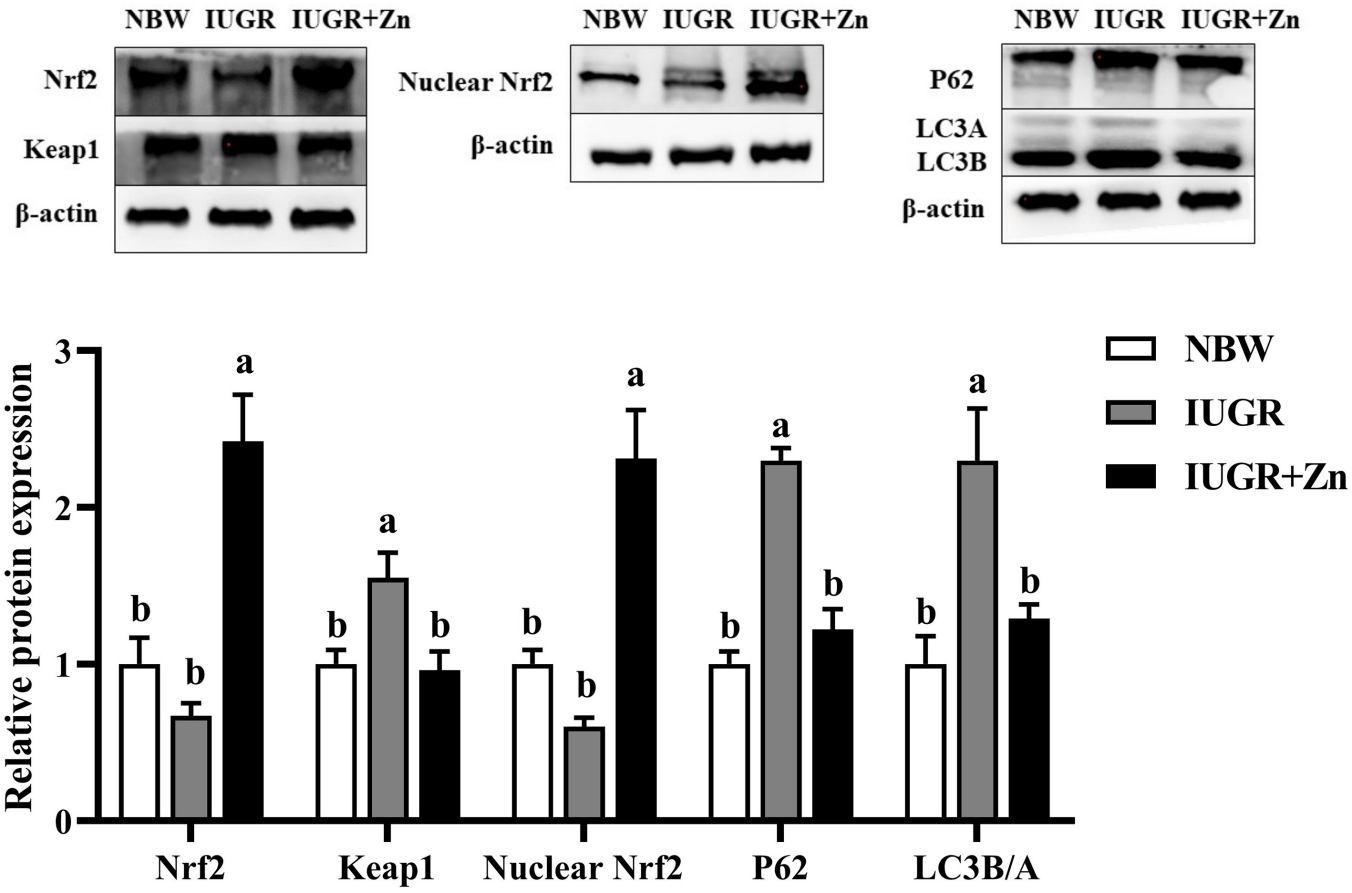
Effects of dietary Nano-ZnO supplementation on the protein expression in jejunal mucosa of IUGR finishing pigs. Data were expressed relative to β-actin and normalized to the NBW group, and expressed as means with their SEM (*n* = 4). ^a, b^Means for the same parameter with different superscripts are significantly different (*P* < 0.05). Nrf2, nuclear respiratory factor 2; P62, Sequestosome 1; LC3B/A, microtubule-associated protein light chain 3 B/A. NBW, normal birth weight pigs; IUGR, intrauterine growth restriction pigs; IUGR+Zn, IUGR pigs fed with diets supplemented with 600 mg Zn/kg from Nano-ZnO.

As shown in Figure 4, compared with the NBW finishing pigs, the protein expressions of Keap1, P62 and LC3B/A were upregulated in the jejunal mucosa of IUGR finishing pigs (*P* < 0.05), while no significant changes in protein expressions of total Nrf2 and nuclear Nrf2 of jejunal mucosa were observed between the NBW and IUGR groups (*P* > 0.05). Dietary added 600 mg Zn/kg from Nano-ZnO significantly decreased the protein expression of Keap1, P62 and LC3B/A, and increased the protein expressions of total Nrf2 and nuclear Nrf2 in jejunal mucosa of IUGR finishing pigs as compared with the IUGR group (*P* < 0.05). Pigs in IUGR+Zn group had significantly enhanced the protein expression of total Nrf2 and nuclear Nrf2 as compared with the NBW group (*P* < 0.05), and no significant differences in protein expression of Keap1, P62 and LC3B/A were observed (*P* > 0.05).

## Discussion

Many studies have reported that IUGR can lead to low birth weight in piglets, but the body weight of IUGR pigs will show a catch-up in growth and can be equal to that of normal pigs (8, 9, 27). In this study, IUGR reduced the body weight and ADG of piglets at 1 d and 21d, while IUGR pigs had no significant difference in body weight (at 77 and 163 d), and in ADG (during 21 to 77 d and 77 to 163 d) as compared to the NBW group, suggesting that IUGR pigs showed catch-up growth, which is in agreement with the previous studies (8, 9, 27). Dietary supplementation with 600 mg Zn/kg from Nano-ZnO had no significant effects on the body weight and ADG of IUGR pigs, but the catch-up growth was not observed. This might be partially due to the decreased ADFI (during 77 to 163 d) by the dietary 600 mg Zn/kg from Nano-ZnO. In line with our present study, Sandoval et al. (28) and Miller et al. (29) found that high dietary zinc could decrease the feed intake of animals, which is related to the duration, dosage and bioavailability of zinc sources (30). These results indicated that more researches might be needed to further explore the appropriate duration and dosage of dietary Nano-ZnO in IUGR pigs, especially for the fattening stage.

One important reason for the inhibited growth of IUGR pigs might be the lack of circulating concentration of IGF-1, which is mainly regulated by GH (31–33). Plasma IGF-1 concentration of the IUGR piglets was about 30–35% lower than the NBW piglets (34), while increasing IGF-1 level (*via* injection or gene therapy) could improve the growth and development of IUGR animals (35, 36). In our present study, there was no significant difference in the serum GH and IGF-1 concentration between NBW and IUGR groups, which is in line with the similar body weight at 77 and 163 d between these two groups. Moreover, dietary 600 mg Zn/kg from Nano-ZnO also did not significantly affect the serum GH and IGF-1 concentration of IUGR pigs, which is in agreement with no significant effect on the growth of IUGR pigs. However, there was also no significant difference in serum GH and IGF-1 between NBW and IUGR+Zn groups, suggesting that the reason for pigs in IUGR+Zn group did not show catch-up growth might be related to other reasons instead of circulating IGF-1 concentration.

The intestinal tract is an important organ for digestion, absorption and immunity. The VH, VW, CD, VH/CD and VSA are critical indicators for evaluating intestinal morphology, and nutrient absorption capacity (37, 38). Many studies support the idea that damage occurs to the intestinal morphology and integrity in piglets with IUGR (19, 20). Interestingly, in the present study, we observed that IUGR increased the jejunal VH, CD, and VSA of the finishing pigs, indicating that IUGR finishing pigs achieved improved jejunal morphology during the fattening stage, which might improve their digestion and absorption abilities. We speculated that the improvement in the jejunal morphology may be related to the later catch-up growth of the IUGR pigs. The Nano-ZnO supplementation significantly decreased CD and increased the VH/CD radio of the IUGR finishing pigs, indicating that Nano-ZnO could further improve the IUGR pig jejunal intestinal morphology. This is consistent with our previous results of the effects of Nano-ZnO on the intestinal morphology of weaned piglets (16, 18).

The content of IgG, IgM and IgA in animals can reflect the state of immune response and can be used to diagnose immune diseases. Many studies have reported decreased immunoglobulin content and induced inflammatory reactions in the serum and intestine of IUGR piglets (20, 21, 39). In our study, no significant difference was observed in the contents of IgG, IgM and IgA in the serum or jejunal mucosa between the NBW and IUGR groups, indicating restoration of the immunoglobulin levels in IUGR pigs during the fattening period. IgA in mucosal tissues is the first line of defense for preventing the invasion of harmful bacteria from invading the body and it is involved in intestinal mucosal inflammation. Dietary Nano-ZnO reduced IgA content in the jejunal mucosa but did not change any other immune indexes. Similar results were found in piglets by Cui et al. (40) who reported that the IgA content in the jejunum of piglets decreased with the increase in the levels of supplemental Nano-ZnO in the feed (maximum 500 mg/kg). We suspect that the antibacterial properties of Nano-ZnO may inhibit the immune stress caused by intestinal harmful bacteria, thereby reducing the IgA content in intestinal mucosa (41, 42).

We further investigated the intestinal inflammatory response induced by IUGR and the beneficial effects of dietary Nano-ZnO supplementation by examining the expression of inflammation-related mRNA in jejunal mucosa. IL-4 and IL-10 are anti-inflammatory cytokines, and IL-1β, IL-6, TNF-α and IFN-γ are pro-inflammatory cytokines (43). TNF-α can bind to the corresponding receptors and activate the NF-κB signaling pathway, resulting in the release of other pro-inflammatory cytokines (e.g., TNF-β, IL-1, IL-2), and triggering of the inflammatory response (44). In the present study, the up-regulation of mRNA expression of IL-6, TNF-α and NF-κB suggested that IUGR-induced inflammatory responses may still exist in jejunal mucosa of finishing pigs. Importantly, the expression of TNF-α and NF-κB mRNA were decreased, whereas the expression of IL-10 mRNA was increased by dietary 600 mg Zn/kg from Nano-ZnO, suggesting that Nano-ZnO can inhibit the activation of the NF-κB signal in jejunal mucosa of IUGR finishing pigs, and thereby alleviate the inflammatory response of the jejunal mucosa. Long et al. (43) also reported that Nano-ZnO has a significant anti-inflammatory effect on weaned piglets, while Xia et al. (45) reported that dietary Nano-ZnO significantly reduced the relative mRNA expression levels of IFN-γ, IL-1β, TNF-α and NF-κB in jejunum of piglets. These observations support the possibility that IUGR-affected finishing pigs fed a diet supplemented with Nano-ZnO might mitigate jejunal mucosa inflammation by transcriptional regulation of TNF-α, NF-κB, and IL-10 expression.

Many studies have confirmed that IUGR can induce oxidative stress in piglets, including oxidative stress in serum (46), leg muscle (9), longissimus dorsi muscle (47), liver (48), and intestinal tract (20). Oxidative stress is caused by the imbalance between the antioxidant system and peroxides in the organisms, and eventually leads to lipid peroxidation and protein oxidation due to excessive production of reactive oxygen species (ROS) in the tissues. The main markers of lipid peroxidation and protein oxidation are MDA and PC, respectively (49, 50). The T-AOC activity and GSH content can reflect non-enzymatic antioxidant system, GSH is a polypeptide that can eliminate peroxides and also serve as the active substrate of GSH-Px. CAT, T-SOD, and GSH-Px are all important antioxidant enzymes in the antioxidant system of organisms and can reflect enzymatic antioxidant systems. In the current study, we observed that the IUGR finishing pigs had increased concentrations of MDA and PC, and that the levels of the CAT, T-AOC, T-SOD, GSH-Px, and GSH were restored to the normal level of jejunal mucosa, indicating that IUGR-induced oxidative stress still exists in the jejunum of pigs during the fattening stage, but the antioxidant capacity was partly improved. Fortunately, supplementation with Nano-ZnO could alleviate the oxidative stress in the jejunum of IUGR finishing pigs by reducing the content of MDA and PC and by increasing the CAT, GSH-Px and T-AOC activity, and the GSH contents. Nano-ZnO has potent antioxidant power both in cell-free systems and under intracellular conditions (51). In agreement with the trend of our results, Bai et al. (52) found that adding Nano-ZnO to the diet could enhance the antioxidant capacity of growing pigs. Zhao et al. (53) reported that adding appropriate concentration of Nano-ZnO to diets can improve the antioxidant capacity and reduce MDA production of broilers.

The Nrf2/Keap1 signaling pathway is one of the most important mechanisms for resistance to oxidative stress damage. Nrf2 can regulate the expression of antioxidant genes and induce the production of related antioxidant proteins. Nrf2 separated from Keap1 protein in the cytoplasm can enter the nucleus to induce the expression of related antioxidant genes and protect the tissue from oxidative stress damage (54–56). HO-1, GCLC, GCLM, SOD1 and GPx1 are common downstream genes activated by Nrf2 in the antioxidant system, and their expression can improve the antioxidant capacity of tissues (57). IUGR has been confirmed to inhibit the gene and protein expression of Nrf2 and to down-regulate the expression of related antioxidant genes of the intestinal tract in piglets (58). In the current study, we saw significantly upregulation of Keap1 protein expression in the jejunal mucosa of IUGR pigs, indicating that IUGR induced oxidative stress in the jejunal mucosa by promoting expression of Keap1 protein. However, Nano-ZnO upregulated the expression of GCLM and GPx1 by decreasing the Keap1 expression, and increasing the Nrf2 expression and protein level in the nucleus, which was consistent with the results of the antioxidant-related enzyme activities. A recent study in rainbow trout reported that dietary supplementation of Nano-ZnO upregulated the expression of SOD1, SOD2, CAT and GPx1 by increasing Nrf2 gene expression (59), and we saw the same effect on IUGR finishing pigs. These results suggested that Nano-ZnO may enhance the antioxidant capacity of IUGR pigs by promoting Nrf2/Keap1 signal pathway in jejunal mucosa, improving the activity of related antioxidant enzymes, and reducing the generation of MDA and PC to alleviate oxidative stress.

Mitochondria play an important role in cellular respiration, but they are also the main sites of energy metabolism and endogenous reactive oxygen species (ROS) production (22). Oxidative stress still existed in the jejunal mucosa of IUGR finishing pigs, which may further cause changes in mitochondrial structure. Mitochondrial swelling is a hallmark of mitochondrial dysfunction and is one of the most important indicators of the opening of the mitochondrial permeability transition (MPT) pore (60). Oxidative stress can affect the activity of related mitochondrial respiratory chain complexes, and the MPT pores may be further activated, greatly increasing the probability of pore opening, aggravating the loss of mitochondrial matrix components, and ultimately leading to mitochondrial swelling (61, 62). Autophagy is an organism's defense mechanism in response to oxidative stress by removing damaged proteins and organelles, while over-autophagy may further induce apoptosis or tissue damage (63, 64). Excessive ROS can lead to mitochondrial damage, which further leads to selective autophagy of damaged mitochondria (65). P62 is a multifunctional ubiquitin-binding scaffold protein that has a variety of cellular functions ranging from cell signaling to autophagy (66). LC3 is a marker of autophagy and is mainly involved in the formation of autophagosomes. It is a structural protein of the autophagosome, and its C-terminal 5-peptide is cut off by ATG4B and lysed to form the cytoplasmic form LC3A, which is then activated by APG7L/ATG7 and transferred to ATG3 and coupled with lipoacylethanolamine to form the membrane-bound form, LC3B, which can be attached to the membranes of autophagosomes, and further form autolysosomes with the lysosome (67, 68). The ratio of LC3B to LC3A can reflect the degree of autophagy. When oxidative stress occurs, P62 increases in number and aggregates, and P62 helps identify autophagy substrates through its ubiquitin-binding domain and promotes interaction with LC3 (69). The results of the present study showed that the number of swollen mitochondria and autolysosomes, and the protein expressions of P62 and ratio of LC3B to LC3A were increased in the jejunum of IUGR finishing pigs, and mitochondria were mostly vacuolated and disorganized. These results suggested that IUGR enhanced the jejunal mucosal autophagy of pigs. Similarly, previous studies have shown that IUGR increases the number of abnormal mitochondrial and autophagy protein expression in the intestinal mucosa of piglets (20, 70). However, dietary supplementation with Nano-ZnO reduced the number of swollen mitochondria and autolysosomes, improved the shape and structure of mitochondria and down-regulated the protein expression of P62 and the ratio of LC3B to LC3A in jejunal mucosa of IUGR finishing pigs, suggesting that Nano-ZnO could reduce IUGR-induced autophagy in jejunal mucosa. This is consistent with our above results that Nano-ZnO might alleviate jejunal oxidative stress through the Nrf2/Keap1 regulatory pathway.

## Conclusion

Our results indicated that IUGR pigs showed postnatal catch-up growth and improved intestinal morphology in the fattening stage, but still existed oxidative stress and inflammation in jejunum. Dietary supplemental 600 mg Zn/kg from Nano-ZnO may further improve intestinal morphology, reduce inflammatory response, decrease autophagy and alleviate oxidative stress *via* enhancing the Nrf2/Keap1 signaling pathway in jejunum of IUGR pigs.

## Data Availability Statement

The author selected the following statement: The datasets presented in this study can be found in online repositories. The names of the repository/repositories and accession number(s) can be found below: https://www.ncbi.nlm.nih.gov/.

## Ethics Statement

The animal study protocol was approved by the Institutional Animal Care and Use Committee of Nanjing Agricultural University (Jiangsu, China. Permit number SYXK-2019-00142).

## Author Contributions

CW and TW conceived and designed the experiments. BZ, JZ, HL, and SC prepared material and collected data. BZ analyzed the data and prepared and drafted the manuscript. CW and BZ revised the manuscript. All authors have read and agreed to the published version of the manuscript.

## Funding

This study was financially supported by the National Natural Science Foundation of China (No.31972598).

## Conflict of Interest

The authors declare that the research was conducted in the absence of any commercial or financial relationships that could be construed as a potential conflict of interest.

## Publisher's Note

All claims expressed in this article are solely those of the authors and do not necessarily represent those of their affiliated organizations, or those of the publisher, the editors and the reviewers. Any product that may be evaluated in this article, or claim that may be made by its manufacturer, is not guaranteed or endorsed by the publisher.
